# Longitudinal Dissociation of the Left Bundle Branch: Two Different Patterns of S‐V Isoelectric Interval

**DOI:** 10.1002/joa3.70394

**Published:** 2026-06-29

**Authors:** Weilin Chen, Lishan Lin, Longfu Jiang

**Affiliations:** ^1^ School of Medicine Ningbo University Ningbo Zhejiang China; ^2^ Department of Cardiovascular Medicine Ningbo NO. 2 Hospital Ningbo Zhejiang China

**Keywords:** left bundle branch pacing, longitudinal dissociation, selective left bundle branch pacing

## Abstract

S‐LBBP reveals two distinct intracardiac electrogram patterns and divergent V6 RWPT at different pacing outputs. This finding confirms the existence of left bundle branch longitudinal dissociation in clinical pacing practice.

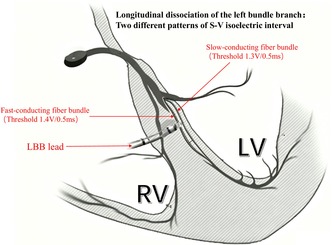

During the implantation of the left bundle branch pacing electrode in an 82‐year‐old male patient with high‐grade atrioventricular block, combined with left anterior fascicular block and right bundle branch block, we observed a simultaneous increase in V6 RWPT (R‐Wave Peak Time: The interval from pacing stimulus onset to the R‐wave peak) at the moment the S‐V (pacing stimulus to V potential) isoelectric interval emerged (Intracardiac recordings were acquired using the Abbott EP Workmate system with a sweep speed of 100–150 mm/s, high‐pass filtering at 200 Hz and low‐pass filtering at 500 Hz. Under these settings, unipolar pacing intracardiac electrograms (EGMs) were recorded at a gain of 0.5 mV/cm to measure discrete intracardiac intervals equivalent to the smooth S‐V interval. Ventricular current of injury (COI) was monitored using a 0.5–500 Hz band‐pass filter with an amplitude calibration of 4 mV/cm. During lead implantation, continuous tip‐cathode unipolar pacing was delivered at 1.5 V/0.5 ms until stable left bundle branch (LBB) capture was confirmed over two consecutive beats). Additionally, two distinct intracardiac electrogram patterns of S‐V isoelectric interval were recorded (Figure 1A), indicating differential capture of left bundle branch subdivisions. These variations stemmed from heterogeneous excitation thresholds among left bundle branch components, resulting in two distinct modes of Purkinje fiber‐mediated conduction propagation within the myocardium, which ultimately manifested as divergent V6 RWPT. Furthermore, during the subsequent threshold testing process (tip unipolar), S‐V dissociation of S‐LBBP (Selective left bundle branch pacing) was observed at both 1.4 and 1.3 V, but with two distinct EGMs and V6 RWPT (72 ms vs. 117 ms), suggesting that at a lower output, one branch of the left bundle branch induced capture loss and switched to another branch with a lower threshold for conduction (Figure 1B), providing robust evidence for longitudinal dissociation of the left bundle branch.

**FIGURE 1:**
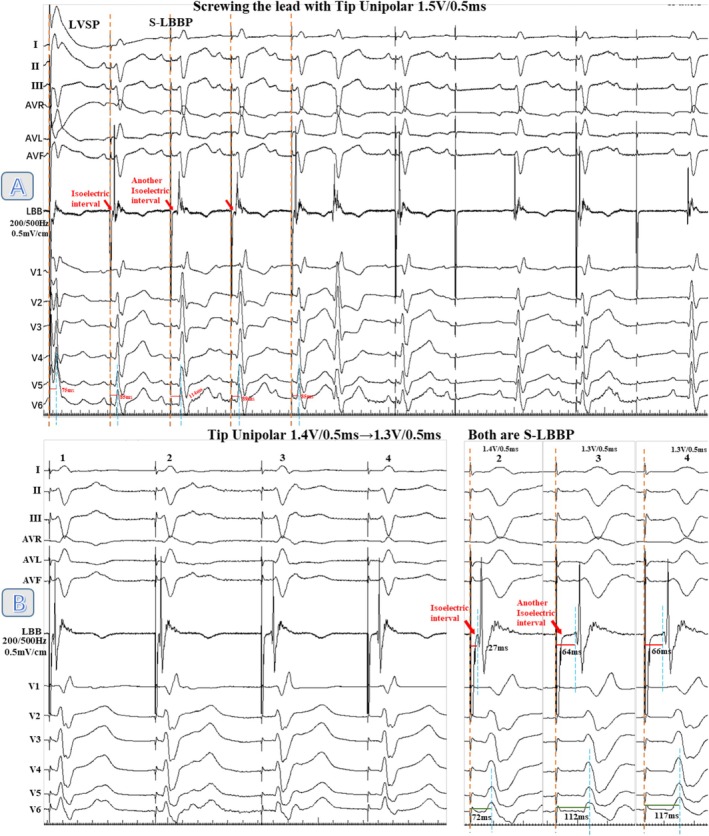
(A) Continuous recording of S‐V isoelectric interval during electrode screwing, showing two morphologies of selective left bundle branch pacing with distinct V6 RWPT and S‐V smooth interval. (B) Two different patterns of S‐V isoelectric interval reappeared during threshold testing. At 1.4 V/0.5 ms, the V6 RWPT was 72 ms, while it prolonged to 112 ms at 1.3 V/0.5 ms. Both pacing outputs yielded consistent selective left bundle branch capture, accompanied by distinct morphological manifestations in other surface ECG leads.

## Funding

This work was supported by the Ningbo Major Research and Development Plan Project (grant number 2024Z235), the Project of NINGBO Leading Medical & Health Discipline (grant number 2026‐A29), the Medical Scientific Research Foundation of Zhejiang Province, China (grant number 2025KY1403), and the Zhu Xiu Shan Talent Project of Ningbo No. 2 Hospital, China (grant number 2023HMYQ18).

## Conflicts of Interest

The authors declare no conflicts of interest.

## Supporting information


**Figure S1:** No LBB Potential in native EGM at the target position.
**Figure S2:** Different ECG morphologies appear during different S‐V dissociation EGM morphologies' existence.

**Video S1:** Intracardiac electrograms and 12‐lead ECG of patient during pacing.

## Data Availability

The data that support the findings of this study are available on request from the corresponding author. The data are not publicly available due to privacy or ethical restrictions.

