# Supplementary material for: Longitudinal Dissociation of the Left Bundle Branch: Two Different Patterns of S‐V Isoelectric Interval
Source: J Arrhythm. 2026 Jun 29;42(4):e70394. doi: 10.1002/joa3.70394 (PMC13312801; doi:10.1002/joa3.70394)

Supplementary Figure 1: No LBB Potential in native EGM at the target position.

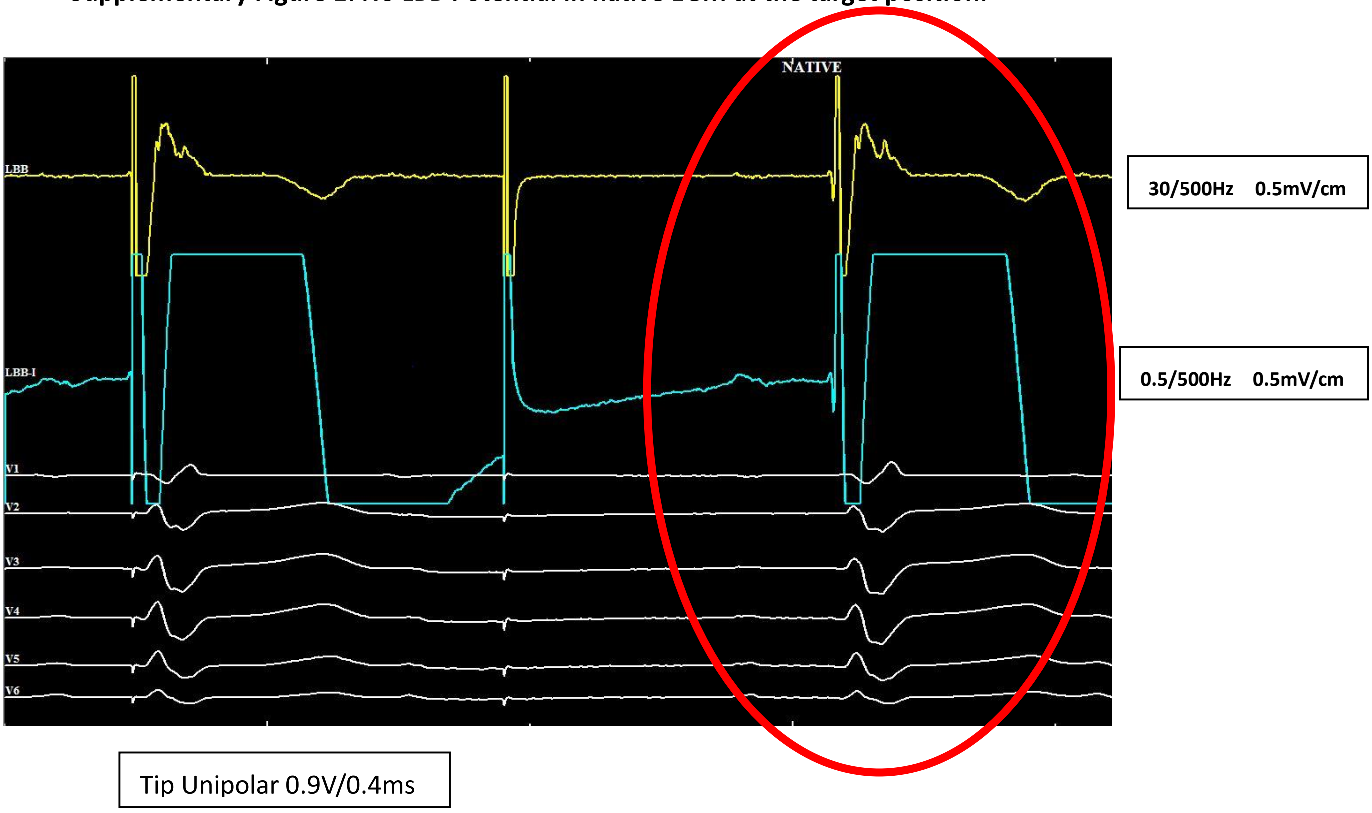

Supplementary Figure 2: Different ECG morphologies appear during different S-V dissociation EGM morphologies' existence.

27/72/141MS means that In the intracardiac LBB lead, the interval from the stimulus (S) to the onset of the V wave was 27ms, and the V6 RWPT was 72ms, V1 RWPT was 141ms.

The label TD 1.3 V in the figure is an incorrect marking. The correct annotation should be Tip Unipolar 1.3 V, as indicated at the bottom of the figure.

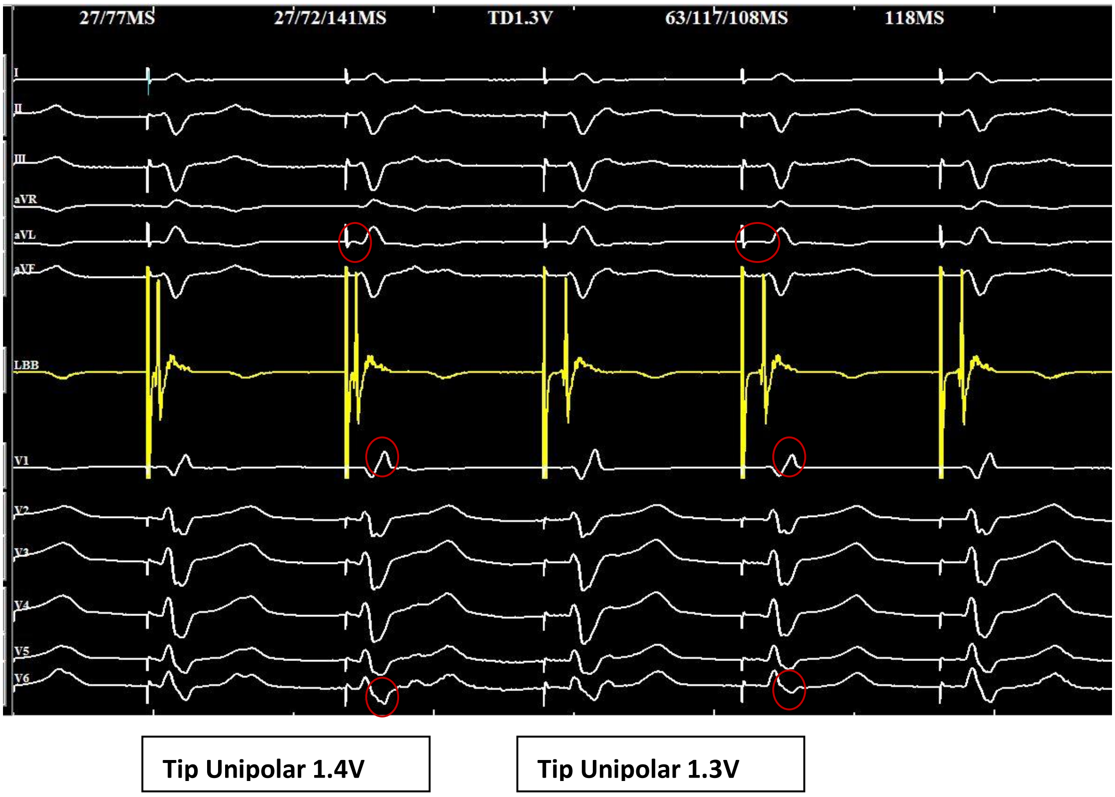

Supplement: Supplementary file 1 — Figure S1: No LBB Potential in native EGM at the target position. Figure S2: Different ECG morphologies appear during different S‐V dissociation EGM morphologies' existence. [file JOA3-42-e70394-s002.pdf]
